# Benchmark of screening markers for KEAP1/NFE2L2 mutations and joint analysis with the K1N2-score

**DOI:** 10.1038/s41698-024-00744-1

**Published:** 2024-11-11

**Authors:** Christoph Arolt, Andreas H. Scheel, Margaret Dugan, Robert Wild, Vanessa Richartz, Barbara Holz, Johannes Brägelmann, Svenja Wagener-Ryczek, Sabine Merkelbach-Bruse, Juergen Wolf, Reinhard Buettner, Luigi Catanzariti, Matthias Scheffler, Axel M. Hillmer

**Affiliations:** 1 Institute of Pathology, Faculty of Medicine and University Hospital Cologne, University of Cologne, Cologne, Germany; 2 Dracen Pharmaceuticals Inc., San Diego, CA USA; 3 Center for Molecular Medicine Cologne, University of Cologne, Cologne, Germany; 4 Department of Translational Genomics, University of Cologne, Faculty of Medicine and University Hospital Cologne, Cologne, Germany; 5 Mildred Scheel School of Oncology, University of Cologne, Faculty of Medicine and University Hospital Cologne, Cologne, Germany; 6 Lung Cancer Group Cologne, Center for Integrated Oncology Cologne/Bonn, University Hospital Cologne, Cologne, Germany; 7 Department I for Internal Medicine, Center for Integrated Oncology Cologne/Bonn, University Hospital Cologne, Cologne, Germany

**Keywords:** Non-small-cell lung cancer, Cancer genomics

## Abstract

Our recently published K1N2-score robustly predicts *KEAP1/NFE2L2*-mutations and pathway activation status, while its accessibility might be limited. We tested if the RNA expression data of six pathway-related genes and NQO1-IHC might be a reliable alternative using 348 *KEAP1/NFE2L2* mutation-enriched NSCLC. While *TXNRD1* RNA testing was the best-performing single-gene test, the combination of single-gene screening and validation with the K1N2-score achieved the highest performance when predicting mutation status or pathway activation.

Mutations of Kelch-like ECH-associated protein 1 (*KEAP1*) or Nuclear factor erythroid 2-related factor 2 (*NFE2L2/NRF2*) lead to an activated KEAP1/NFE2L2 pathway in NSCLC, resulting in a discrete tumor phenotype with poor overall outcome and relative resistance to chemotherapy^1,2^. However, this pathway can potentially be therapeutically targeted^3–6^. We recently published a transcriptomic 46-gene signature, the K1N2 score, that robustly predicted *KEAP1/NFE2L2* mutation status but also outperformed mutation testing with respect to survival and tumor hypoxia prediction^7^. Moreover, it was capable of detecting alterations in other genes that lead to an activated pathway. Thus, it represents a tool that could potentially select NSCLC patients for pathway-directed targeted therapy. Following the scientific discourse that was prompted by our study^8,9^, we anticipated that the implementation of the K1N2 score would probably be restricted to large tertiary care centers that provide NanoString assays in clinical routine. Consequently, we aimed to explore simpler surrogates of KEAP1/NFE2L2 pathway activation such as monogenetic RNA expression tests or immunohistochemistry (IHC).

NAD(P)H:Chinonoxidoteduktase-1 (*NQO1*), is one of the many NFE2L2 targets that plays a role in ameliorating intracellular oxidative stress in solid tumors^10^. Romero et al. recently proposed NQO1 IHC as a potential surrogate for *KEAP1* mutations and pathway activation in *KEAP1* defective genetically engineered mice and human NSCLC. In two well conducted studies, they experimentally identified *NQO1* as a target gene for NFE2L2. Also, this group found a significant correlation between *NQO1* RNA expression and *KEAP1/NFE2L2* mutations. However, the 88 human NSCLC that Romero and colleagues analyzed contained only 10 and 2 *KEAP1*- and *NFE2L2*-mutant NSCLC, respectively^3,11^. Regarding our previous publication and the established cohort with enrichment for *KEAP1/NFE2L2* mutant cases, we intended to systematically assess the value of NQO1 IHC to predict *KEAP1/NFE2L2* mutations and the pathway status.

In the present study, we used the previously published Cologne cohort of 348 *KEAP1/NFE2L2* mutation-enriched NSCLC to assess the capability of NQO1 IHC and a selection of monogenetic single-gene RNA tests to predict *KEAP1/NFE2L2* mutations and the KEAP1/NFE2L2 pathway activation as defined by the K1N2 score. We identify several single-gene RNA expression tests, which hold the potential to screen patients for KEAP1/NFE2L2 pathway-directed therapy and could also be carried out in laboratories without access to NanoString equipment.

To assess whether NQO1 IHC would be a reliable predictor for *KEAP1/NFE2L2* mutation status, we stained and semi-quantitatively assessed 232 NSCLC of the Cologne cohort for which enough residual FFPE tissue was available (Fig. 1A). As expected, expression of NQO1 protein was significantly upregulated in mutated LUAD and LUSC (Wilcoxon test; Fig. 1B, C). While NQO1 IHC performed well in validation cohort LUSC (ROC-AUC: 0.845, CI = 0.499–1; sensitivity: 0.810; specificity: 0.875, Fig. 1E), its predictive power was poor in LUAD (ROC-AUC: 0.682, CI = 0.486–0.857; sensitivity: 0.714; specificity: 0.531, Fig. 1D).

**Fig. 1:**
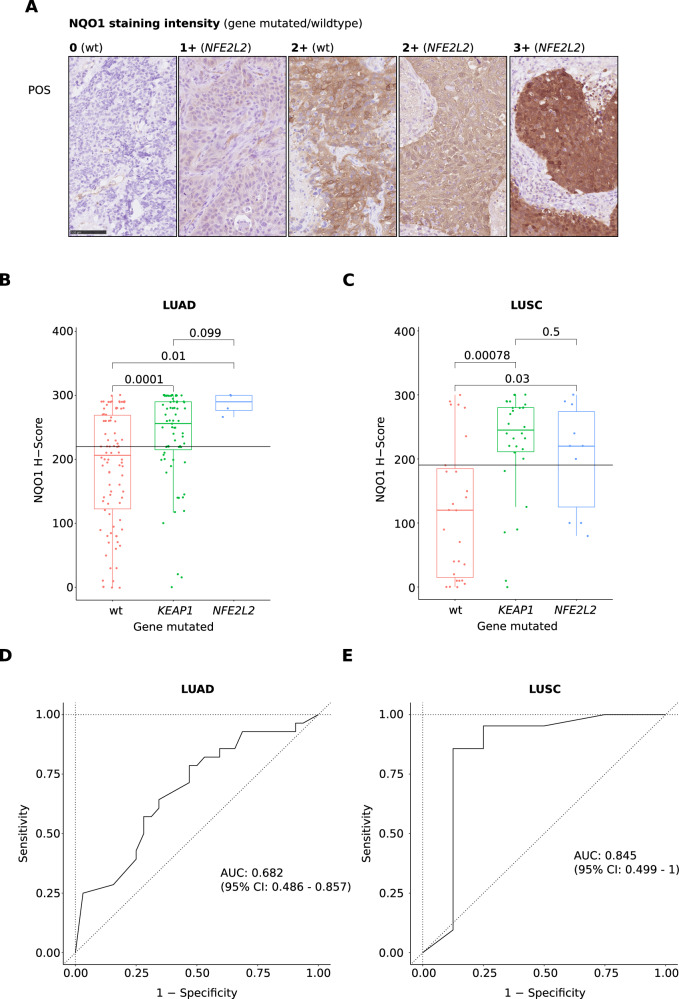
NQO1 protein expression in LUAD and LUSC (IHC). Exemplary NQO1 IHC images with absent (0), mild (1+), moderate (2+), and strong (3+) staining intensities, 400× magnification, 100 μm scalebar (**A**). NQO1 H-score in *KEAP1*mut, *NFE2L2*mut, and wildtype of LUAD (**B**) and LUSC (**C**), pooled analysis of the Cologne_use and the Cologne_validation cohorts. AUC curve of *KEAP1/NFE2L2* mutation prediction using NQO1 IHC in the Cologne validation dataset of LUAD (**D**) and LUSC (**E**).

To evaluate other more accessible methods, we used the NanoString-based RNA expression data that we generated previously to develop the K1N2-score. The five most important coefficient genes from the K1N2-score and *NQO1* were selected. We considered two scenarios for *KEAP1/NFE2L2* mutation status prediction through RNA expression data in clinical practice: (1) to use it as sole test for mutation prediction or (2) to use it as screening test with optimized sensitivity which would be followed by the more expensive and thoroughly validated K1N2-score (combinatorial approach; Fig. 2A). When predicting the *KEAP1/NFE2L2* mutation status, three combinatorial approaches (K1N2 score + *NQO1* RNA / *TXNRD1* RNA / *TRIM16* RNA; Fig. 2B) outperformed all other testing regimes in LUAD, LUSC and the overall cohort (LUAD + LUSC), followed by the original K1N2 score as single test. Again, the overall performance was higher in LUSC than in LUAD with an advantage of ~7–9% in sensitivity/specificity. A combination of *NQO1* RNA succeeded by the K1N2-score for validation in case of a positive screening result (scenario 2) achieved the highest performance in LUAD and the pooled cohort, while its performance in LUSC was identical to screening with *TRIM16* and *TXNRD1* RNA testing. The best single RNA-test in LUSC and the overall cohort was *TXNRD1*, which encodes Thioredoxin Reductase 1. This target of NFEL2L2 is involved in counteracting oxidative stress^12,13^. Of note, *TXNRD1* was the most significant predictor of short survival in a study that analyzed the expression of 64 oxidative stress-related genes in 35 NSCLC datasets^14^. The best single-gene test for LUAD was *NQO1* RNA but it outperformed *TXNRD1* only by <1% sensitivity/specificity.

**Fig. 2:**
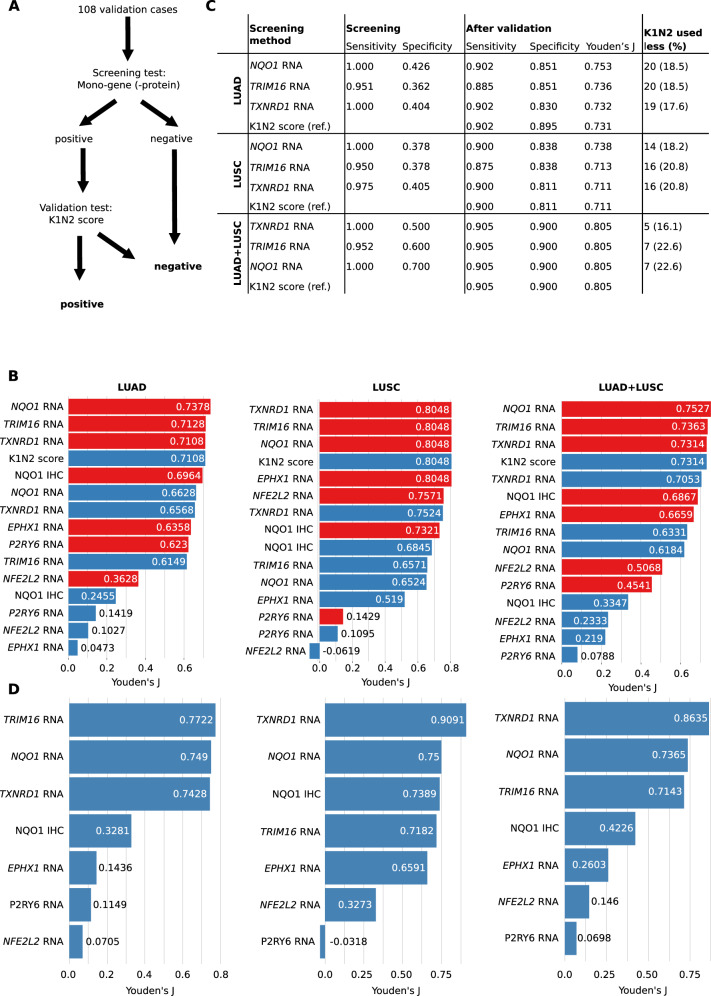
Benchmark of screening markers for *KEAP1/NFE2L2* mutations and KEAP1/NFE2L2 pathway activation. Testing algorithm of the combinatorial approaches using the Cologne validation dataset (**A**). Youden’s J values of single and combinatorial tests predicting the *KEAP1/NFE2L2* mutation status for LUAD, LUSC, and the overall validation cohort (**B**). Detailed results of the best-performing combinatorial tests with the K1N2 score as reference; screening and validation refer to (**A**) indicating high sensitivity and low specificity at the screening step and high sensitivity and specificity after K1N2-score validation test of the positive screening cases (**C**). Youden’s J values for single-gene tests when predicting KEAP1/NFE2L2 pathway activation status as defined by the K1N2 score in LUAD, LUSC, and the overall validation cohort (**D**).

We recently demonstrated that the K1N2 score robustly predicts the *KEAP1/NFE2L2* mutation status in NSCLC. However, these results indicate that its performance can even be increased by combining it with single-gene RNA assays (*NQO1*, *TRIM16*, *TXNRD1*). Additionally, introducing a screening test can potentially be more cost efficient (Fig. 2C). Even though all cases would have to be screened, the number of cases that would undergo expensive K1N2 testing with a 46-gene NanoString panel could be reduced by 16.1–22.6% (Fig. 2C).

As detailed in our previous report^7^, the K1N2 score outperforms *KEAP1/NFE2L2* mutation testing when predicting tumor hypoxia and patient prognosis. Also, it is capable of detecting other pathway-activating genetic alterations. Since these findings might justify using the K1N2 score not as a tool to predict mutational status but as a new gold standard KEAP1/NFE2L2 pathway activation surrogate, we also tested how well mono-gene tests predict the pathway status as defined by the K1N2 score (Fig. 2D). mRNA expression of *TXNRD1* outperformed all other tests including NQO1 IHC in LUSC and the overall cohort, reaching an excellent Youden’s J of 0.91 (equivalent to a sensitivity and specificity of ~ 95%) in LUSC and a Youden’s J of 0.86 in the pooled dataset. In LUAD, it was only marginally outperformed by *TRIM16* and *NQO1* RNA testing. As expected, NQO1 IHC performed relatively well for LUSC but was clearly outperformed by *TXNRD1* RNA testing.

Here, we systematically benchmark the potential of RNA expression of five selected KEAP1/NFE2L2 pathway-related genes as well as *NQO1* RNA to predict *KEAP1/NFE2L2* mutation and pathway activation status as defined by our recently published K1N2 score. We also test the predictive power of NQO1 IHC which was proposed as a surrogate test, recently^3^. Similar to the K1N2-score, these tests hold the potential to directly identify an upregulation of the KEAP1/NFE2L2 pathway irrespective of the underlying DNA mutation. We have recently shown that mutations in *CUL3* and *SMARCA4* can also cause a KEAP1/NFE2L2 pathway activation^7^. A test for KEAP1/NFE2L2 pathway activation is clinically relevant since targeted inhibitions of this pathway might become treatment options in the future^3–6^.

With 115 out of 232 samples harboring *KEAP1/NFE2L2* mutations, this is by far the largest cohort of mutated NSCLC tested with NQO1 IHC. We find that NQO1 IHC performs well in LUSC with much poorer performance in LUAD. The overall moderate predictive power of the assay might in part be attributable to the heterogenous intra-sample expression of this protein in NSCLC^15^ which principally complicates IHC scoring. Surprisingly, NQO1 IHC is consistently outperformed by RNA expression testing of *TXNRD1* and *NQO1* when predicting *KEAP1/NFE2L2* mutation status alone or in combination with the K1N2 score or when predicting KEAP1/NFE2L2 pathway activation as defined by the K1N2 score. Moreover, the combination approaches involving single-gene screening tests for *TXNRD1, TRIM16,* and *NQO1* RNA expression and K1N2-score as validation outperformed all single tests including the K1N2 score alone. Most testing regimes performed better in LUSC than LUAD, while still achieving a maximum Youden’s J of 0.74 for LUAD which is equivalent to a sensitivity/specificity of ~87%.

Combinatorial testing not only increased the precision but also holds the potential to reduce cost. Moreover, the single-gene tests might be a valuable tool to assess or pre-screen mutation/pathway-activation status when a suitable NanoString infrastructure is not available. For instance, screened, positive cases might secondarily be sent to tertiary care structures for validation with the K1N2 score. This concept of specialized centers for molecular testing in NSCLC has recently been found to prolong survival of cancer patients in Germany^16^. However, we acknowledge that this combined approach might be reserved for cases with sufficient FFPE tissue available, such as resection specimens.

In our previous report, we provided evidence that a gene expression test such as the K1N2 score holds the potential to detect KEAP1/NFE2L2 pathway alterations more precisely than the mutational status of pathway related genes such as *KEAP1*^7^. In the present study, we suggest several single gene tests that achieve a Youden’s J of up to 91% when predicting the pathway status as defined by the K1N2 score. Ultimately, all these predictive genomic, transcriptomic and proteomic tests will require thorough evaluation alongside clinical trials that target the KEAP1/NFE2L2 pathway which we hope has been made more feasible through our efforts.

## Methods

### Immunohistochemistry

232 NSCLC were available for NQO1 IHC. Staining with anti-NQO1 (Cell Signaling Technology, US-MA, clone D6H3A) was carried out with a Leica BOND-MAX stainer (Leica Biosystems, Wetzlar, Germany). The staining intensity was semi-quantitatively and jointly measured by two pathologists (AC, CA) and a consensus H-Score (range 0-300) was noted^17^.

### RNA expression analysis and test strategy

Furthermore, the NanoString RNA expression values of the five most important coefficients (five most important signature genes) of the K1N2-score from all 348 patients of the Cologne cohort were used^7^. To assess the predictive power of the mentioned assays, we divided the cohort into a Cologne-use (RNA tests: 173 LUAD, 67 LUSC; IHC: 105 LUAD, 38 LUSC) and a Cologne-validation cohort (RNA tests 77 LUAD, 31 LUSC; IHC: 60 LUAD, 29 LUSC) as described before^7^. The former was used to optimize a threshold, while the latter independent dataset was used to assess the test performance. Additional splitting of the cohort into LUAD and LUSC specimens was carried out in case of histology-specific tests. For single-assay tests, the cutoff was optimized for Youden’s J (= sensitivity + specificity – 1). When used as screening test, the cutoff was optimized to maximize the sensitivity at a minimum specificity of 0.3. The K1N2-score predictions and performance values were taken over from our prior publication^7^. The combined testing approach consisted of screening with a single gene RNA or IHC test followed by K1N2 score testing in case of a positive screening result. All tests except the original K1N2 score were separately trained and optimized for LUAD, LUSC, and the overall cohort to maximize test performance.

### Statistical programs and ethics

All statistical analyses were performed in *R* programming language. Cutoffs were optimized using the *cutpointr R* package, and performance values were calculated as described before^7^. All analyses were conducted according to local ethical guidelines were reviewed by the ethics committee of the University of Cologne (reference number 10–242). All participants gave their informed consent to participate.

## Data Availability

All NanoString gene expression data used here is available as Supplement to our previous article^7^. The NQO1 IHC data is available upon request.

## Code availability

No custom code was used.
